# 3D Microfluidic Bone Tumor Microenvironment Comprised of Hydroxyapatite/Fibrin Composite

**DOI:** 10.3389/fbioe.2019.00168

**Published:** 2019-07-17

**Authors:** Jungho Ahn, Jungeun Lim, Norhana Jusoh, Jungseub Lee, Tae-Eun Park, YongTae Kim, Jangho Kim, Noo Li Jeon

**Affiliations:** ^1^Department of Mechanical and Aerospace Engineering, Seoul National University, Seoul, South Korea; ^2^George W. Woodruff School of Mechanical Engineering, Georgia Institute of Technology, Atlanta, GA, United States; ^3^Faculty of Engineering, School of Biomedical Engineering and Health Sciences, Universiti Teknologi Malaysia, Johor Bahru, Malaysia; ^4^Ulsan National Institute of Science and Technology, Ulsan, South Korea; ^5^Parker H. Petit Institute for Bioengineering and Bioscience, Georgia Institute of Technology, Atlanta, GA, United States; ^6^Institute for Electronics and Nanotechnology, Georgia Institute of Technology, Atlanta, GA, United States; ^7^Wallace H. Coulter Department of Biomedical Engineering, Georgia Institute of Technology, Atlanta, GA, United States; ^8^Department of Rural and Biosystems Engineering, Chonnam National University, Gwangju, South Korea; ^9^Division of WCU (World Class University) Multiscale Mechanical Design, Seoul National University, Seoul, South Korea; ^10^Seoul National University Institute of Advanced Machines and Design, Seoul, South Korea; ^11^Institute of Bioengineering, Seoul National University, Seoul, South Korea

**Keywords:** tumor microenvironment, vascularized tumor, cancer metastasis, angiogenesis, hydroxyapatite, fibrin matrix, microfluidic platform

## Abstract

Bone is one of the most common sites of cancer metastasis, as its fertile microenvironment attracts tumor cells. The unique mechanical properties of bone extracellular matrix (ECM), mainly composed of hydroxyapatite (HA) affect a number of cellular responses in the tumor microenvironment (TME) such as proliferation, migration, viability, and morphology, as well as angiogenic activity, which is related to bone metastasis. In this study, we engineered a bone-mimetic microenvironment to investigate the interactions between the TME and HA using a microfluidic platform designed for culturing tumor cells in 3D bone-mimetic composite of HA and fibrin. We developed a bone metastasis TME model from colorectal cancer (SW620) and gastric cancer (MKN74) cells, which has very poor prognosis but rarely been investigated. The microfluidic platform enabled straightforward formation of 3D TME composed the hydrogel and multiple cell types. This facilitated monitoring of the effect of HA concentration and culture time on the TME. In 3D bone mimicking culture, we found that HA rich microenvironment affects cell viability, proliferation and cancer cell cytoplasmic volume in a manner dependent on the different metastatic cancer cell types and culture duration indicating the spatial heterogeneity (different origin of metastatic cancer) and temporal heterogeneity (growth time of cancer) of TME. We also found that both SW620 and MKN72 cells exhibited significantly reduced migration at higher HA concentration in our platform indicating inhibitory effect of HA in both cancer cells migration. Next, we quantitatively analyzed angiogenic sprouts induced by paracrine factors that secreted by TME and showed paracrine signals from tumor and stromal cell with a high HA concentration resulted in the formation of fewer sprouts. Finally we reconstituted vascularized TME allowing direct interaction between angiogenic sprouts and tumor-stroma microspheroids in a bone-mimicking microenvironment composing a tunable HA/fibrin composite. Our multifarious approach could be applied to drug screening and mechanistic studies of the metastasis, growth, and progression of bone tumors.

## Introduction

Cancer metastasis, a complex phenomenon in which cancer cells spread to new organs, is one of the greatest challenges in cancer research. Metastasis is influenced by surrounding stromal cells and the extracellular matrix (ECM), as well as tumor cells (Coleman, 2001). Tumor angiogenesis also contributes to cancer metastasis (Weis and Cheresh, 2011; Bielenberg and Zetter, 2015). Therefore, tumor angiogenesis and the interactions between cancer cells and the ECM influence the invasion and metastasis of cancer cells (Ma et al., 2008; Gill and West, 2014). To investigate the complex interactions in the tumor microenvironment (TME), previous studies have constructed *in vitro* TME models (Wang et al., 2014; Ahn et al., 2017; Chung et al., 2017), which led to the development of a drug/nanoparticle delivery test system for cancer therapy (Ahn et al., 2018a; Du et al., 2018; Sei et al., 2018).

Bone is one of the most common locations of cancer cell metastasis. Aggressive tumor cells are prone to be extremely metastatic in the fertile bone environment (Coleman, 2001), becoming a frequent cause of morbidity and mortality in patients with advanced cancer (Coleman, 2001; Pathi et al., 2010). The prognosis of patients with bone metastasis is very poor, with a 5-year survival rate of <5%. Most patients at stage 3 or 4 have bone metastasis (>51% for colorectal cancer and >61% for gastric cancer) at the initial diagnosis of colorectal and gastric cancer (Ahn et al., 2011; Baek et al., 2016). To recapitulate bone metastasis from colorectal and gastric cancer, we used the highly metastatic colon cancer cell line SW620 (from a lymph node metastasis) and the gastric cancer cell line MKN74 (from a liver metastasis). The prognosis of patients with bone metastasis from colorectal and gastric cancer is poor because of the advanced stage of the disease and largely unknown metastatic behavior in the bone TME (Sundermeyer et al., 2005). The bone microenvironment consists of the inorganic mineral hydroxyapatite [Ca_10_(PO_4_)_6_(OH)_2_] (HA), with a thickness of 1.5–4.5 nm. HA is located within mineralized fibrils and has high stiffness, toughness, and biocompatibility (Lai et al., 2014). Thus, the addition of HA nanoparticles to the ECM enables recapitulation of the physiological environment *in vitro* (Pathi et al., 2010, 2011; Zhu et al., 2016b). Moreover, due to the unique properties of HA, synthetic HA nanoparticles have been used for biomedical applications (Hanawa, 2012) to promote cell growth and inhibit apoptosis (Shi et al., 2009).

The effects of bone mineral components on tumor cells have been investigated. In addition to enhancing normal bone formation, the HA-mineralized microenvironment mediates bone cancer metastasis and, consequently, secondary tumor formation due to increased cellular adhesion and proliferation, and anti-inflammatory activity (Pathi et al., 2010, 2011; Pilmane et al., 2011; Zhu et al., 2016b). Although HA nanoparticles exert minimal effects on normal cells, they inhibit the proliferation of tumor cells, such as breast cancer, colorectal cancer, gastric cancer, and hepatoma cells (Liu et al., 2003; Yin et al., 2006; Chen et al., 2007; Alépée et al., 2010; Meena et al., 2012; Dey et al., 2014; Han et al., 2014). Furthermore, the inhibitory effect of HA nanoparticles on cancer cells is dose-dependent (Han et al., 2014). However, the cause of metastasis in the bone microstructure is unclear.

The effects of the mechanical properties of the ECM on cells can be analyzed by observing cellular mobility in an HA/hydrogel composite. For example, the effect of an increased concentration of HA in the HA/collagen/fibrin composite on periosteal and endothelial cells (ECs) has been examined *in vivo* and *in vitro* (Zhang et al., 2006; Rao et al., 2014). Moreover, bone metastasis of breast and prostate cancer cells was recapitulated *in vitro* using an HA-containing scaffold (Lin et al., 2001; Meena et al., 2012). However, rheological analysis of HA-containing ECM, which affects cellular mobility, is lacking. Furthermore, an *in vitro* model of cancer metastasis in an osseous microenvironment focused on breast and prostate cancer (Lin et al., 2001; Pathi et al., 2011; Zhu et al., 2016a). Microfluidic devices have been developed to investigate breast cancer metastasis and cancer extravasation in an osteocell microenvironment (Bersini et al., 2014). Compared to breast and prostate cancer, bone metastasis of gastric cancer (Yoshikawa and Kitaoka, 1983; Kammori et al., 2001; Ahn et al., 2011) and colon cancer (Katoh et al., 1995; Sundermeyer et al., 2005) rarely delays diagnosis.

**Figure 1:**
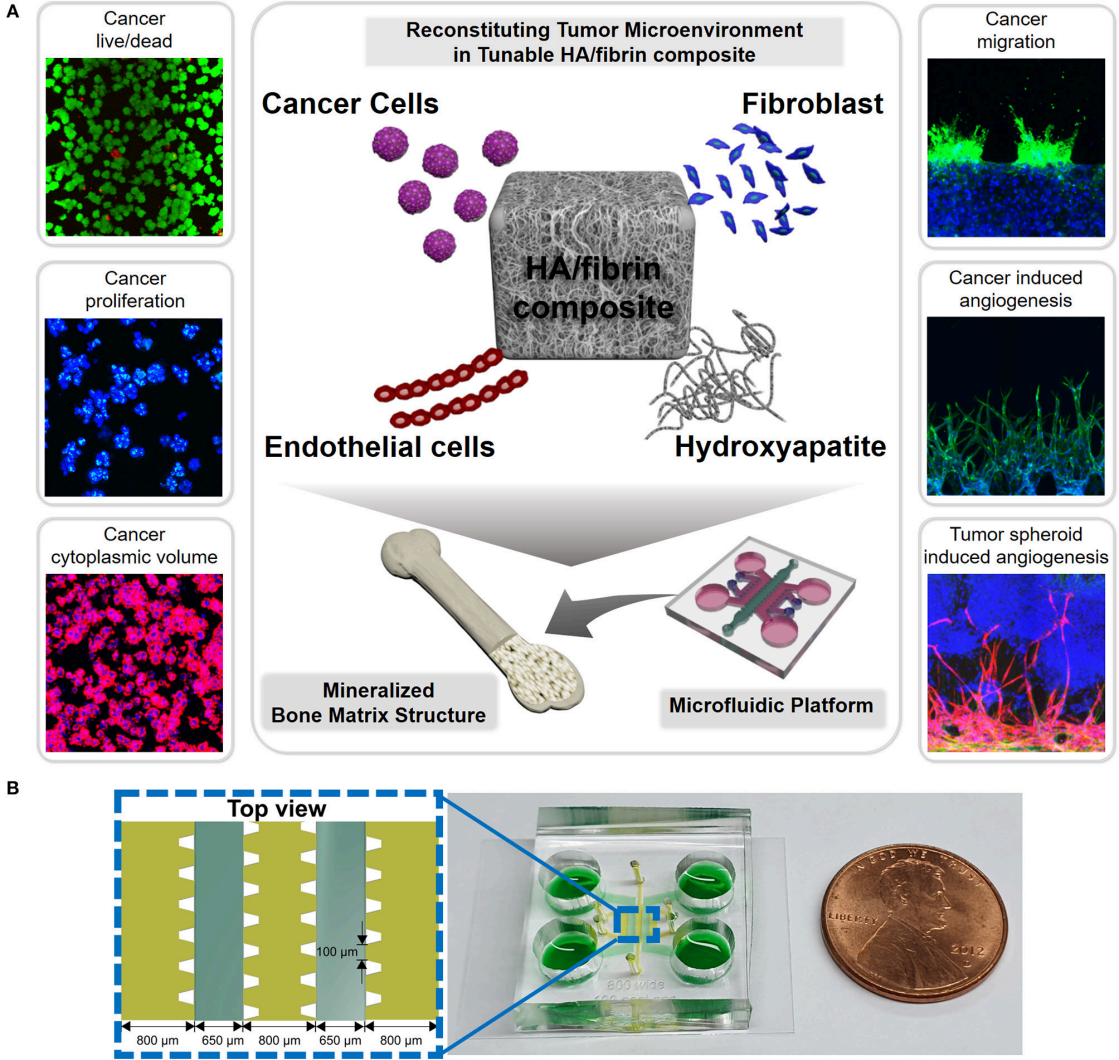
Scheme for reconstruction of tumor microenvironment (TME) in bone-mimicking extracellular matrix (ECM) composite in the microfluidic chip. **(A)** The composites include cells composing of tumor microenvironment such as cancer cells, endothelial cells and fibroblasts as well as hydroxyapatite (HA) mineral component consisting of bone matrix. Using this microfluidic platform, we analyzed cancer viability, proliferation, cytoplasmic volume, migration, cancer induced angiogenesis, and tumor spheroid induced angiogenesis. **(B)** Configuration of microchannels of the microfluidic platform indicating their dimensions and a photograph of a microfluidic chip for bone-mimicking TME.

In this study, we analyzed the gel rheology of an HA/fibrin composite to assess the influence of the mechanical properties of the ECM on the TME (Figure 1). We incorporated colorectal cancer (SW620) and gastric cancer (MKN74) cells within a bone-mimicking microfluidic platform consisting of an HA/fibrin composite and investigated their viability, morphology, proliferation, and migration. Fibroblasts were co-cultured for the bone microenvironment instead of osteoblasts, as the fibroblasts within the bone matrix are genetically analogous to osteoblasts (Ducy et al., 2000). Furthermore, tumor angiogenesis, a key factor in cancer metastasis, was investigated using our platform by assessing sprouting characteristics and secreted factors. Moreover, the vascularized TME was reconstructed in the bone-mimicking matrix, which included three-dimensional (3D) tumor spheroids that resembled the TME in bone. By reconstituting bone microstructure with the TME in a microfluidic platform and analyzing its rheological properties of the microstructure, we evaluated the relationship between the properties of the ECM and tumor angiogenesis and metastasis.

## Results and Discussion

### Morphology of the HA/Fibrin Composite

To recapitulate the mineralized bone TME, which comprises mainly a stiff ECM and calcium phosphate (which provide hardness and rigidity), we incorporated HA into the fibrin ECM. We have reported previously on various structures consisting of pure fibrin (0% HA) and fibrin with 0.05, 0.10, 0.30, 0.40, and 0.50% HA (Jusoh et al., 2015). In our bone TME platform, HA particles are distributed homogenously within a fibrin gel and can be readily observed. We confirmed that at ≥0.5% HA, the fibrin-HA mixture does not polymerize in the microfluidic channel (Jusoh et al., 2015). Therefore, we used fibrin/HA concentrations of 0.0, 0.2, and 0.4%.

**Figure 2:**
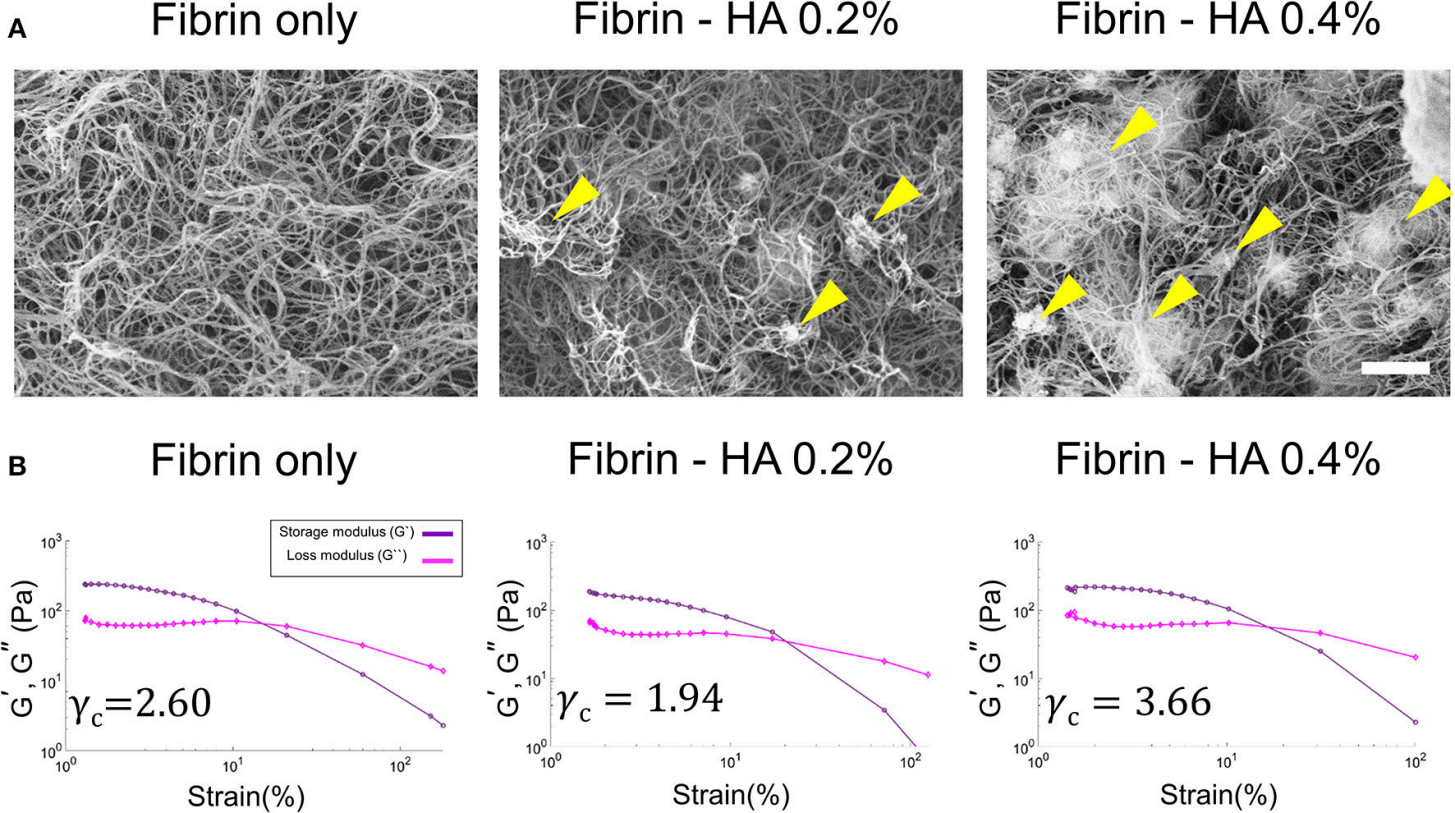
**(A)** SEM images of HA/fibrin composite with varying concentration of HA component (0.0, 0.2, and 0.4%). (Scale bar: 2 μm). Yellow arrows indicate agglomerated locations. **(B)** The storage and loss modulus as a function of strain at the same temperature and oscillation frequency in gel rheology analysis, indicating critical strain for each plot.

A scanning electron microscopy (SEM) image of the HA/fibrin composite containing 0.0, 0.2, and 0.4% HA is shown in Figure 2A. As the concentration of HA increased, the locations showing agglomeration thereof within the HA/fibrin composite also increased. Additionally, while the fibrin gel had a relatively uniform inter-fiber pore size, the pore size of the HA/fibrin composite was distributed non-homogeneously. Moreover, the composites with higher concentrations of HA were characterized by larger gaps between agglomerated HA nanocomposites. In contrast, as the concentration of HA increased, the number of gaps between agglomerated HA nanocomposites decreased, because the total amount of agglomerated HA nanoparticles increased to fill the gaps. Furthermore, when the HA/fibrin composite was prepared in six-well plates (Figure S1A), the time required for it to solidify increased with increasing HA concentration (Figure S1B).

### Mechanical Properties of the HA/Fibrin Composite

The mechanical properties (including the storage and loss moduli, as determined by gel rheology under strain sweep conditions) of the HA/fibrin composites with varying concentrations of HA are shown in Figure 2B, Figure S2. The critical strain (γ_c_) was defined as the strain above which the storage modulus decreased by more than 10% of the maximum value of the storage modulus (Mancini et al., 2002). The critical strain value of the fibrin gel was significantly lower at 0.2% HA (γ_c_ = 1.94) compared to 0.0% HA (γ_c_ = 2.60). In contrast, the critical strain was lower at 0.4% HA (γ_c_ = 3.66) compared to 0.2% HA. In addition, under 5% oscillation strain, the storage modulus was lower at 0.2% HA (G' = 122 Pa) compared to 0.0% HA (G' = 166 Pa), but was higher at 0.4% HA (G' = 174 Pa) compared to 0.2% HA.

Previous studies have addressed the relationship between tissue morphology/response and the mechanical properties of the gel composite (Rao et al., 2012, 2014). As noted above, the HA/fibrin composite was subjected to varying strain waves in gel rheology testing to analyze the critical strain (γ_c_), which represents the strain zone in which the modulus deviates from linear behavior. Both the critical strain and the storage/loss modulus at 5% oscillation strain were lower at 0.2% HA compared to the conditioned fibrin gel, but higher at 0.4% HA (Figure S3). Therefore, the mechanical properties of the HA/fibrin composite were affected by the quantity of HA, possibly due to effect on the morphology and architecture of the matrix.

### An HA/Fibrin Composite for Recapitulation of the TME

The microfluidic device consisted of five parallel channels separated by 100-μm gaps. To prevent leakage and to capture the hydrogels, microposts were used in the microfluidic device (Figure 1). By keeping HA nanoparticles in the fibrin ECM, this platform allows detailed analysis of the HA-TME interaction, which modulates cellular activity (Figure 1). Note that an ideal platform for engineering bone-mimetic ECM should have suitable 3D structures with interconnected pores to facilitate cellular activities, while also supporting cell adhesion, proliferation, and differentiation (Shamloo and Heilshorn, 2010).

As a scaffolding biomaterial, collagen-hydroxyapatite composite scaffold was characterized for bone tissue engineering (Rodrigues et al., 2003). Fibrin also has been widely known as a biopolymer to emulate the natural nanostructured features of bone and be favorable to precisely control its nano/microstructure (Noori et al., 2017). Since collagen demonstrates similar characteristics to fibrin in terms of fiber thicknesses, the level of the total serum protein adsorption, and bone regenerative response, both biomaterials have been frequently used in bone tissue engineering (Oh et al., 2014). Thus, a plethora of studies in bone tissue engineering are conducted using both collagen and fibrin composites.

### Tumor Cell Viability, Proliferation, and Morphology

**Figure 3:**
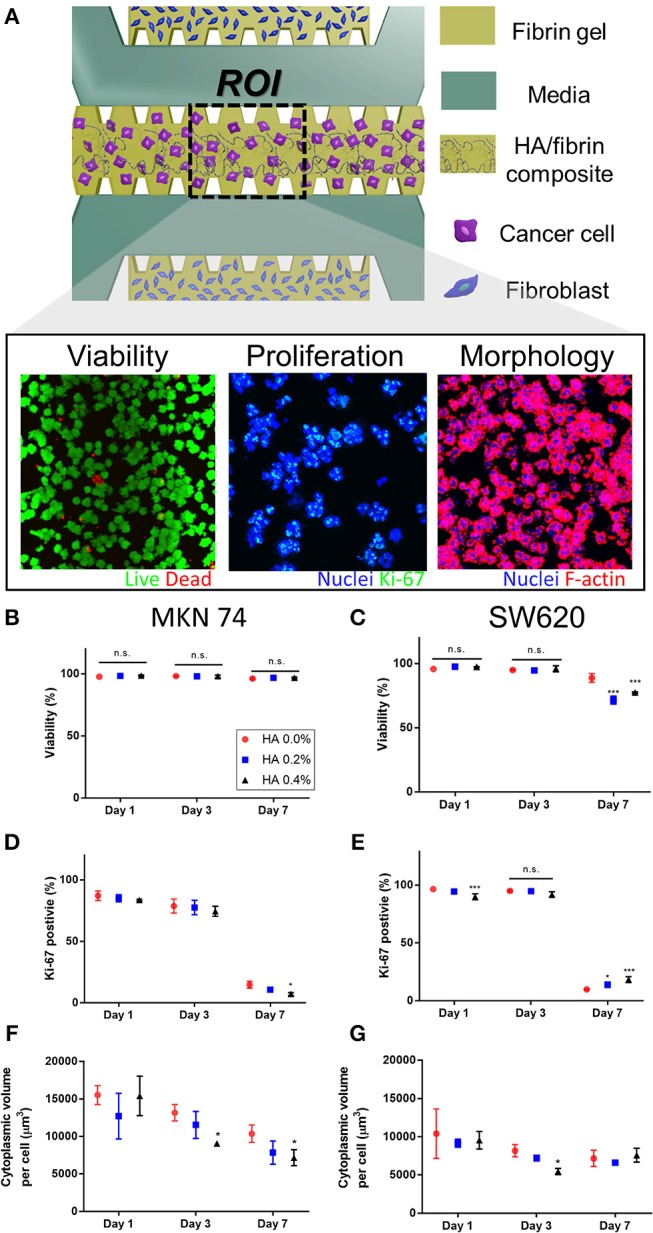
Cancer cellular response assay in the HA/fibrin composite in the microfluidic platform **(A)** Schematic representation of assay to observe cellular responses of cancer cells cultured in the HA/fibrin composite in TME co-cultured with fibroblasts. Representative confocal images of viability, proliferation, and morphology of cancer cells. Quantification of assay for **(C,D)** viability, **(D,E)** proliferation, **(F,G)** morphology conducted in the microfluidic chip in different HA concentration and culture time points. (**B,D,F**: MKN 74, and **C,E,G**: SW620, *n* = 4–6 chips per condition) The representative confocal Images are demonstrated in Figures S4, S6, S8, respectively.

SW620 and MKN74 cells were incubated in a HA/fibrin composite with 0.0, 0.2, or 0.4% HA for 1, 3, or 7 days (Figures S4–S9, Figure 3). Cell viability was quantified by live/dead assay, proliferation was assessed by Ki-67 staining, and cell morphology was analyzed by determining the cytoplasmic volume per cell by filamentous actin (F-actin) staining (Figures S4–S9). We patterned tumor cells with 0.0, 0.2, and 0.4% HA/fibrin composite into the middle channel, and injected fibroblasts into the two side channels to facilitate paracrine interactions between the tumor cells and stromal cells.

MKN74 cells exhibited consistent viability during culture in 0.0, 0.2, and 0.4% HA/fibrin composites (Figures 3B,C, Figures S4, S5). The viability of SW620 cells at days 1 and 3 did not differ significantly according to HA concentration (Figure 3C). However, at day 7, the viability of SW620 cells cultured in 0.2 and 0.4% HA was significantly decreased compared to those in 0.0% HA. In two-dimensional (2D) culture, the viability of SW620 cells was not significantly different, but that of MKN74 cells decreased with increasing HA concentration (Figure S5). The proliferation rate (number of Ki-67 positive cells) of both cell types decreased significantly over time (Figures 3D,E, Figures S6, S7). Interestingly, the number of Ki-67–positive MKN74 cells cultured in 0.4% HA at day 7 was significantly lower than in the control (Figure 3D). The proliferation rate of SW620 cells cultured in 0.4% HA was significantly lower than that of cells cultured in 0.0% HA at day 1, but the opposite trend was evident at day 7 (Figure 3E). In 2D culture, there was no significant difference in the proliferation rate of MKN74 cells, but the proliferation rate of SW620 cells cultured in 0.4% HA was markedly lower than that of the control (Figure S7). The cytoplasmic volume per cell of MKN74, but not SW620 cells, decreased over time (Figures 3F,G, Figures S8, S9). The cytoplasmic volume per cell of MKN74 cells decreased with increasing HA concentration at days 3 and 7, and did that of SW620 cells at day 3 (Figures 3F,G). Interestingly, SW620 cells with 0.4% HA showed a biphasic effect on cytoplasmic volume per cell with increasing culture time (Figure 3G). However, in 2D culture, the cytoplasmic volume per cell of both cell types was unaffected by the HA concentration (Figure S9). Thus, tumor cell proliferation and cytoplasmic volume per cell differed according to the HA concentration in the HA/fibrin composite, and by time, but the trends varied between the two cell lines.

HA components do not significantly affect cell viability, enabling their clinical application; e.g., as doxorubicin-loaded HA-coated nanoparticles in drug delivery systems for targeted cancer therapy (Abbasi Aval et al., 2016). In this study, the viability of SW620 (days 1 and 3) and MKN74 (days 1, 3, and 7) cells remained high in the HA/fibrin bone-like matrix irrespective of the HA concentration, but the viability of SW620 cells at day 7 with a high HA concentration significantly decreased compared to the control, suggesting an anti-tumor effect of HA. Therefore, HA affects cell viability in a manner dependent on the cell type and culture duration. The cytoplasmic volume of both SW620 and MKN74 cells tended to decrease with increasing culture duration irrespective of the HA concentration (Figures 3F,G). The cytoplasmic volume of MKN74 tumor cells tended to decrease with increasing HA concentration at days 3 and 7, but not SW620 cells, likely due to the increased agglomerated locations, which might hinder the volumetric growth of the cytoplasm in tumor cells.

The cancer stroma plays roles in cancer at multiple stages. Indeed, tumor cells do not act in isolation, but rather subsist in an abundant microenvironment nourished by resident stromal cells (e.g., fibroblasts and ECs) and the ECM. The tumor stroma is essential for cancer initiation, growth, and progression (Pietras and Ostman, 2010). In a previous study, fibroblasts induced remarkable morphological changes in SW620 and MKN74 cells within 48 h. Both cell types exhibited a significant increase in cytoplasmic volume and clustered nuclei, possibly due to factors secreted by the fibroblasts. In addition, studies involving co-injection of tumor cells and mesenchymal cells from different sources have demonstrated the importance of stromal fibroblasts for tumor initiation, growth, and metastasis (Camps et al., 1990; Karnoub et al., 2007).

HA exerts an anti-proliferation effect, possibly by inducing apoptosis in certain types of cancer cells. HA nanoparticles inhibit proliferation and induce apoptosis by producing intracellular reactive oxygen species and activating p53, which is responsible for DNA damage and apoptosis (Liu et al., 2003; Chen et al., 2007; Alépée et al., 2010; Meena et al., 2012; Dey et al., 2014), and smaller nanoparticles have a stronger anti-cancer effect (Alépée et al., 2010; Meena et al., 2012; Dey et al., 2014). Interestingly, the inhibitory effects of HA nanoparticles may be cell type-specific, as the inhibitory effect of HA is greater for cancer cells than for normal cells (Han et al., 2014). Consistent with previous research, the proliferation rate of MKN74 cells tended to decrease with increasing HA concentration in this study. In contrast, at day 7, the number of Ki-67–expressing SW620 cells increased with increasing HA concentration; the opposite trend was detected at day 1. Therefore, some types of cancer cell might alter their proliferation in response to culture duration and HA concentration. Also, the correlations of the viability, morphology, and proliferation of tumor cells in the TME with HA concentration can be analyzed using our platform.

### Tumor Cell Migration Within the HA/Fibrin Composite

**Figure 4:**
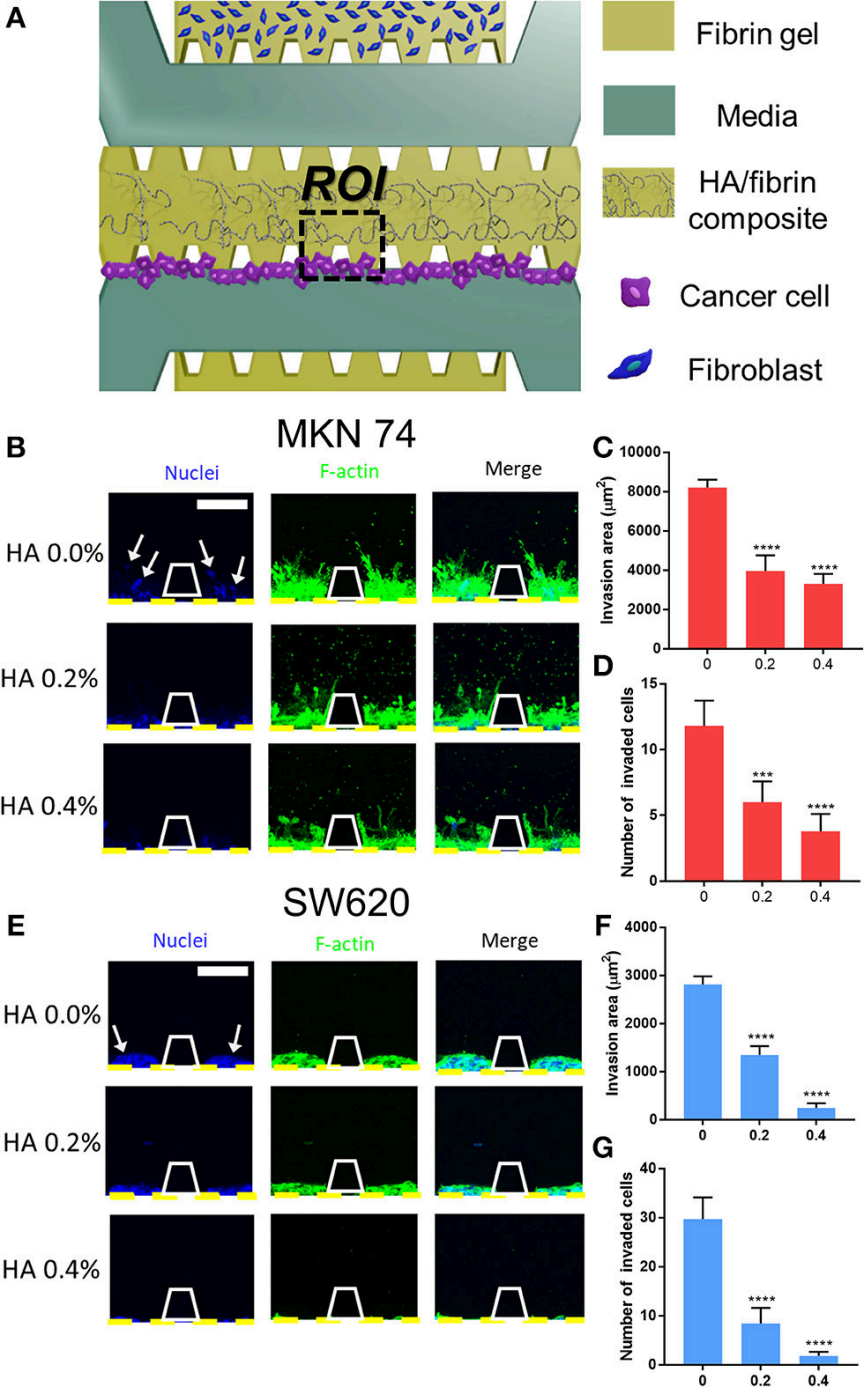
Cancer cell migration in HA/fibrin composite in the microfluidic platform. **(A)** Schematic representation of assay to observe migration of cancer cells cultured in the HA/fibrin composite in TME co-cultured with fibroblasts. Confocal images and graphs for the number of invaded cancer cells and invasion area per ROI for **(B–D)** MKN74, and **(E–G)** SW620 respectively in HA/fibrin composite with varying HA concentration (0.0, 0.2, and 0.4%) (*n* = 5–9 chips per condition) (white arrows indicate nuclei of migrated tumor cell into tunable bone mimicking ECM). (Scale bar: 100 μm).

To evaluate migration of tumor cells through a bone-mimicking matrix, we performed immunostaining for F-actin and confocal microscopy to assess cell morphology (Figure 4). We first attached two types of cancer cells to the side of the middle channel, which contained acellular fibrin gel, and stromal cells plus fibrin gel were patterned in other microchannels to enable paracrine interactions between cancer and stromal cells.

Figures 4C,D,F,G shows quantitative data on the cell invasion of composites consisting of 0.0, 0.2, and 0.4% HA. The HA/fibrin gel construct remained intact under various medium and culture duration conditions (Figure S10).

Generally, the number of invaded cells decreased with increasing HA concentration. In the HA/fibrin composites with 0.0% and 0.2% HA, 11 ± 2 and 6 ± 2 MKN74 cells, respectively, had migrated, fewer than 30 ± 4 and 8 ± 3 SW620 cells, respectively. However, at 0.4% HA, 4 ± 1 MKN74 cells had migrated, compared to 2 ± 1 SW620 cells (Figures 4D,G).

In addition, we quantified the invasion area based on the actin cytoskele-ton. In general, the invasion area of both types of cancer cell decreased with increasing HA concentration. MKN74 cells had a significantly larger invasion area compared to SW620 cells at all HA concentrations tested. For SW620 cells, the invasion area was 2,812 ± 171, 1,344 ± 188, and 244 ± 96 μm^2^ at 0.0, 0.2, and 0.4% HA, respectively. For MKN74 cells, the invasion area was 8,212 ± 403, 3,959 ± 792, and 3,305 ± 506 μm^2^, respectively. Therefore, MKN74 cells exhibited enhanced actin filament formation in the HA/fibrin matrix, despite their less marked migration than SW620 cells (Figures 4C,F).

Cancer progression is regulated by the interaction between tumor cells and the stromal compartment, which consists of stromal cells, ECM, chemokines, and blood vessels (Yamaguchi and Condeelis, 2007). Therefore, cells must degrade and remodel ECM structures to migrate through the ECM (Yamaguchi and Condeelis, 2007). Despite its fertile environment, only certain types of cancer cell preferentially spread to bone (Yoneda and Hiraga, 2005). Therefore, cancer cells with a high predilection for disseminating to bone may have biological properties different from those of cancer cells that rarely spread to bone (Ramaswamy et al., 2002).

It is important to note that both SW620 and MKN74 cells exhibited significantly reduced migration at higher HA concentrations. This was likely because of the increased quantity of agglomerated regions in the microstructure of the HA/fibrin composite, which inhibit cellular movement. These results are in agreement with those of the cytoplasmic volume per cell.

### Angiogenesis Induced by Paracrine Signaling From TME in the HA/Fibrin Composite

**Figure 5:**
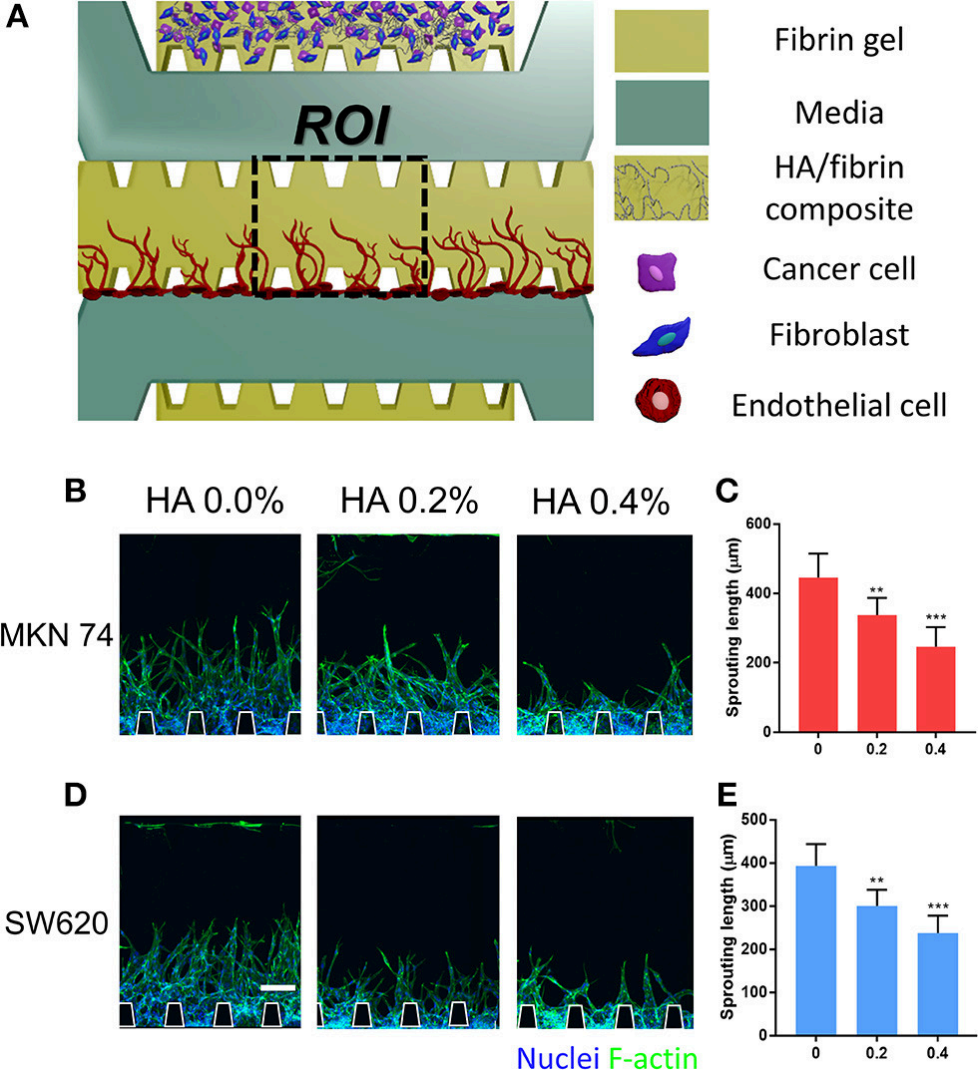
Angiogenesis induced by paracrine signaling from TME co-cultured with cancer cells and fibroblasts in HA/fibrin composite in the microfluidic platform. **(A)** Schematic representation of angiogenesis in fibrin gel, which was induced TME consisting of cancer cells and fibroblasts in bone-mimicking matrix. Confocal image and graph for sprouting length of blood vessels for **(B,C)** MKN74 and **(D,E)** SW620 respectively in HA/fibrin composite with varying HA concentration (0.0, 0.2, and 0.4%). (*n* = 5–9 chips per condition). (Scale bar: 100 μm).

To evaluate TME-induced angiogenesis in the microfluidic platform, cancer cells were incubated with fibroblasts to recapitulate the TME before introducing ECs. Cancer cells and fibroblasts were co-cultured within the HA/fibrin matrix and TME induced-angiogenesis was evaluated (Figure 5A). In our platform, the opening between the posts at the interface of the central channel facilitates paracrine interactions between ECs and cancer/stromal cells during vessel formation. To this end, an acellular fibrin gel was patterned into the middle channel and ECs were attached to the fibrin walls of the middle channel. In general, incubation of MKN74 and SW620 cells with fibroblasts in the HA/fibrin composite reduced angiogenesis, as the sprout length decreased with increasing HA concentration. For MKN74 cells, the sprout length was 445 ± 69, 337 ± 49, and 246 ± 56 μm at 0.0, 0.2, and 0.4% HA, respectively (Figures 5B,C). For SW620 cells, the sprout length was 393 ± 50, 300 ± 37, and 237 ± 40 μm at 0.0, 0.2, and 0.4% HA, respectively (Figures 5D,E). Thus, paracrine signals from tumor and stromal cells in the HA/fibrin composite with a high HA concentration resulted in the formation of fewer sprouts.

The metabolic and nutritional needs for tumor growth and metastasis are met by the formation of a complex angiogenesis vascular network (Alépée et al., 2010; Weis and Cheresh, 2011). Therefore, controlling tumor-associated angiogenesis shows promise for limiting cancer progression (Weis and Cheresh, 2011). *In vivo*, the TME consists of a variety of cell types and numerous signaling molecules and pathways that influence angiogenesis. In addition, tumor cells in the TME activate the surrounding normal cells by releasing cytokines, growth factors, guidance molecules, and matrix metalloproteinases (Alépée et al., 2010; Weis and Cheresh, 2011).

Angiogenesis results from stimulatory signals within the TME that prompt changes in multiple cell types (Weis and Cheresh, 2011). Growth factors and cytokines secreted by tumor cells promote angiogenesis from a nearby vessel for invasion, before intravasating within the local vasculature and, prior to extravasating by adhering to the vessel wall, transmigrating across the endothelium and invading the ECM (Katt et al., 2016). For example, during angiogenesis, vascular endothelial growth factor (VEGF) stimulates the sprouting and proliferation of ECs to form blood vessels (Weis and Cheresh, 2011). By using our platform to enable intercellular communication via secreted factors, we determined that addition of HA to co-cultures of cancer cells and stromal fibroblasts induced secretion of factors that suppressed angiogenesis. Thus, we assayed the concentrations of VEGF, tumor necrosis factor-alpha (TNF-α), and transforming growth factor-beta (TGF-ß) using enzyme-linked immunosorbent assays (Figure S11). The concentrations of these factors differed slightly according to HA concentration. Elucidation of the angiogenic sprouting mechanism may lead to the development of effective therapeutics against angiogenesis in the bone microenvironment.

### Reconstitution of Vascularized Tumor Spheroids in the HA/Fibrin Composite

**Figure 6:**
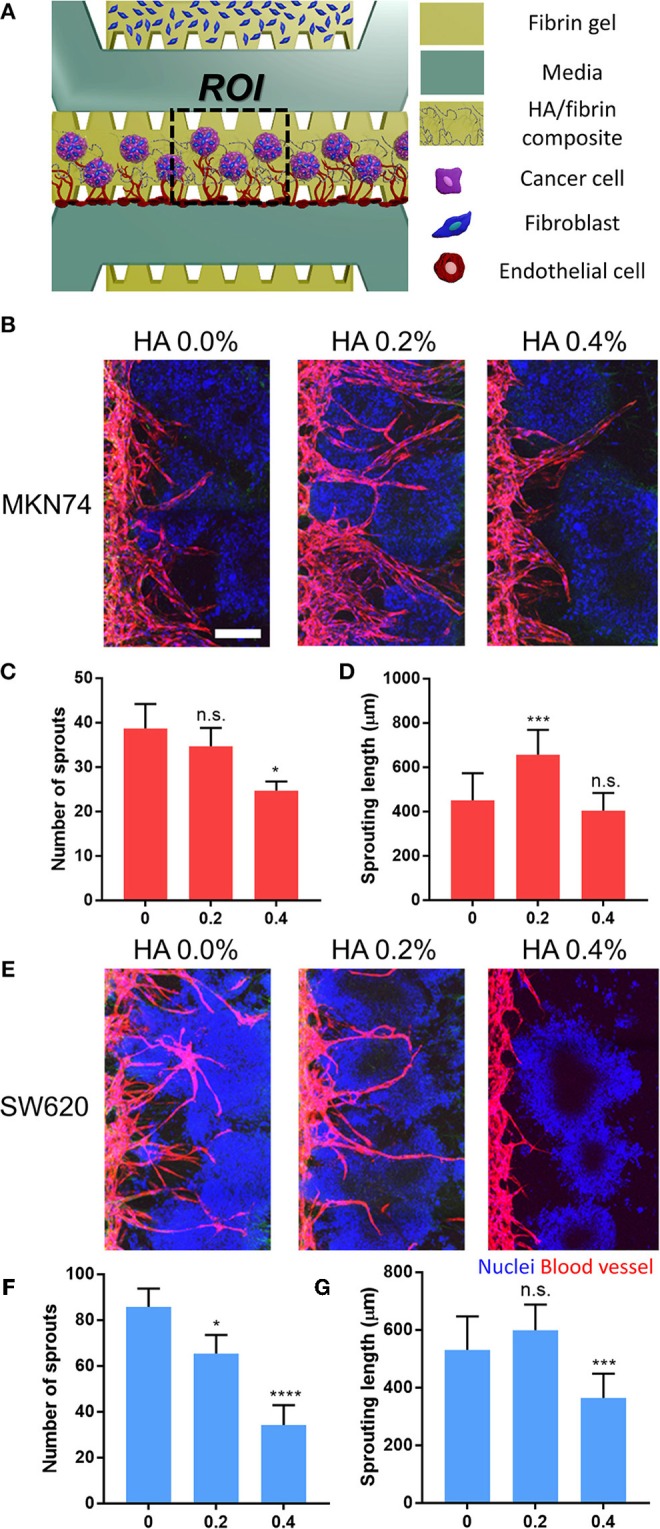
Reconstitution of vascularized three-dimensional tumor-stromal cell spheroid in HA/fibrin composite in the microfluidic platform. **(A)** Schematic representation of assay to observe angiogenesis with three-dimensional tumor spheroid in HA/fibrin composite. Confocal images and graph for the number of blood vessel sprouts and sprouting length of blood vessels for **(B–D)** MKN74 and **(E–G)** SW620 respectively in HA/fibrin composite with varying HA concentration (0.0, 0.2, and 0.4%) (*n* = 5–7 chips per condition). (Scale bar: 200 μm).

To demonstrate crosstalk between the tumor and the TME, we patterned tumor-fibroblast microspheroids into the center channel using HA/fibrin composites comprising 0.0, 0.2, and 0.4% HA (Figure 6). Attached ECs exhibited angiogenic sprouting in response to the microenvironment generated by interactions between SW620- and MKN74-fibroblast microspheroids (Figures 6B,E). For both MKN74 and SW620 cells, the number of angiogenic sprouts decreased with increasing HA concentration (Figures 6C,F). In contrast, sprout length showed a biphasic response to an increasing HA concentration (Figures 6D,G). For MKN74 cells, the sprout length in the 0.2% HA/fibrin composite (656.3 ± 112.6 μm) was dramatically greater compared to the control (450.8 ± 122.4 μm), but did not differ significantly between 0.0% and 0.4% HA. For SW620 cells, the sprout length of the 0.4% HA/fibrin composite (363.9 ± 84.1 μm) was significantly smaller compared to the control (531.1 ± 115.6 μm), and there was no significant difference between 0.0 and 0.2% HA. The new blood vessels physically interacted with the tumor spheroids while migrating through ECM containing bone mineral components, indicating TME-induced angiogenesis (Figures S13, S14, Supplementary Video S1). Interestingly, at 0.4% HA, both MKN74-fibroblast and SW620-fibroblast microspheroid-induced angiogenesis was markedly reduced compared to 0.0% and 0.2% HA, suggesting an anti-angiogenic effect. Importantly, the angiogenesis induced by SW620-fibroblast microspheroids resulted in thinner blood vessels than that promoted by MKN74-fibroblast microspheroids, demonstrating the molecular and cellular heterogeneity of the TME induced angiogenesis.

Within bone environment, cells are steadily encounter physical forces such as hydrostatic pressure, shear, compression and tension. Piezoelectric bone microenvironment can generate electrical signals in response to the mechanical stress (Ribeiro et al., 2015; Hytönen and Wehrle-Haller, 2016; Jacob et al., 2018). These cellular functions involving mechanical interaction are amenable to be modulated by mechanosensitive Piezo channels that transduce mechanical stimuli into intracellular signals (Coste et al., 2010). The balance between mechanical stress and cellular reaction is crucial to sustain tissue homeostasis and, in consequence of loss of the equilibrium leads to pathology including cancer (Michor et al., 2011). Indeed, functional expression of Piezo channels has been demonstrated in malignant breast cancer cell lines (Li et al., 2015). Piezo1 senses rigidity changes in the environment and optimizes confined cell migration (Hung et al., 2016). In addition, Piezo1 served as sensor of shear stress and determinants of vascular structure (Li et al., 2014). Piezo2 is essential to metastatic cancer cells to probe their physical environment as they anchor and pull on their surroundings (Pardo-Pastor et al., 2018). The Piezo2-induced Ca^2+^ signal activates downstream the RhoA-mDia pathway necessary for the regulation of cancer metastasis (Pardo-Pastor et al., 2018). Although in this study focused on the reconstituting of bone-mimicking TME in response to tunable HA concentration, the ability to robustly pattern with multifarious approach could be applied to broad bone TME application including mechanotransduction.

Our platform enables recapitulation of 3D vascularized tumor spheroids in a TME consisting of ECM and minerals. Three-dimensional spheroids comprising tumor fibroblasts formed by aggregation and exhibited protein and mRNA profiles that resembled those *in vivo* (Kloss et al., 2008; Friedrich et al., 2009; Katt et al., 2016). Additionally, the 3D spheroids recapitulated the cell-cell and cell-matrix interactions between tumor cells and the 3D TME (Mehta et al., 2012). Tumor spheroids induce vascularization, which requires a vascular network to deliver oxygen and nutrients (Mueller-Klieser, 1987; Friedrich et al., 2009). In particular, in a bone-like microstructure, the stiffness of the ECM modulates capillary formation and barrier integrity by altering the endothelial response to chemical factors (LaValley and Reinhart-King, 2014).

In TME, pericytes are also capable of tumor homing and crucial stromal cellular components of TME. However, our current system lacked pericytes (von Tell et al., 2006; Ahn et al., 2018b). Therefore, fully mimicking the tumor-stromal interactions in complex bone TMEs remains a challenge.

## Conclusion

As a proof of concept study, we reconstructed the TME in a mineralized microstructure by incorporating highly metastatic SW620 and MKN74 cells within a microfluidic platform. We also investigated the relationship between the mechanical properties and cellular activities of the bone-mimicking ECM. The viability, morphology, and proliferation of both types of cancer cells in the HA/fibrin composite varied according to the HA concentration and culture duration. Moreover, both SW620 and MKN74 cells demonstrated significantly reduced migration at higher HA concentrations. Also, angiogenic sprouts induced by TME paracrine signaling tended to be shorter when ECs were cultured in an HA-rich TME embedded composite. Furthermore, during angiogenesis in tumor-stromal cell spheroids in the HA/fibrin composite, the number of blood vessel sprouts decreased as the HA concentration increased, while the sprout length exhibited a biphasic response to an increasing HA concentration. In conclusion, we established an *in vitro* bone-mimetic TME model within tunable mineralized microstructure, and used it to assess the influence of stromal cells on tumor angiogenesis, migration, and proliferation. The results will promote the development of microfluidic platforms containing physiologically bone-relevant mineralized structures, which will in turn facilitate drug screening and mechanistic studies of tumor metastasis, growth, and progression in an osseous ECM.

## Experimental Section

### Device Fabrication

Microfluidic device with the center channel of 800 μm wide was used in this study. The microfluidic chip were fabricated by polydimethylsiloxane (PDMS, Sylgard 184, Dow Corning) mold embedded with channel structures that was patterned by standard photolithography photoresist SU-8 (MicroChem). Demolded PDMS was punched out by using a biopsy punch (6 mm) and sharpened syringe needle (0.5 mm) to make reservoirs for the medium and hydrogel injection ports. A PDMS device and a glass coverslip was treated with oxygen plasma for 1 min before bringing them into contact. The devices were incubated in an 80°C dry oven for at least 48 h to restore hydrophobicity of PDMS. The devices were sterilized by UV irradiation before use.

### HA/fibrin Composite Preparation

Hydroxyapatite nanocrystals with sizes <200 nm. Hydroxyapatite in EGM-2 media were sonicated for 40 min for particles dilution. The pure fibrin matrix (0.0% HA) was prepared by dissolving 2.5 mg/ml bovine fibrinogen (Sigma) in PBS (Gibco) and supplemented with aprotinin (0.15 U/ml, Sigma) prior mixing with thrombin (0.5 U/ml, Sigma), before left to clot at room temperature for 5 min (Kim et al., 2013). For HA/fibrin composite, the appropriate HA solution (0.0, 0.2, and 0.4% HA) is mixed with the fibrinogen and aprotinin before mixing with the thrombin.

### Mechanical Properties Test

The mechanical properties of acellular HA/fibrin composite were measured by rotational rheometer (TA instrument Ltd., DHR-1, Delaware, USA) equipped with parallel plate (25 mm diameter) at a gap height of 1 mm. The composite with 0, 0.2 and 0.4% HA, respectively was placed on the plate of the rheometer. The storage modulus (G') and loss modulus (G”) were measured using strain sweeping mode with frequency fixed at 1rad/s at 25°C.

### Scanning Electron Microscopy (SEM)

SEM was used as a tool for the observation of the microstructure of HA/fibrin composite. SEM examination of the structure was performed using critical point drying and platinum coating of samples. The composites were first washed in PBS, then fixed in a 2% glutaraldehyde solution for 1 h 30 min. And the composites were washed in 0.05 M sodium cacodylate buffer for 10 min and the washing step is repeated washing step is repeated twice. Fixation was followed by a dehydration series in 30, 50, 70, 80, 90, 100 (× 3) ethanol solutions for 20 min each. Dehydrated samples were placed in a critical point dryer (Leica EM CP300, Germany) and ethanol was replaced with CO_2_ and subsequently removed. The dried samples were mounted on aluminum stubs, sputter-coated with platinum, and examined by field emission scanning electron microscopy (FE-SEM; HITACHI S-5000).

### Cell Cultures

Human umbilical vein endothelial cells (HUVECs, Lonza) and red fluorescent protein-expressing HUVECs (RFP-HUVECs) (Angio-Proteomie) were cultured in endothelial basal medium-2 (EBM-2, Lonza) supplemented with EGM-2 bullet kit and normal human lung fibroblasts (LFs, Lonza) were cultured in fibroblast basal medium (FBM-2, Lonza) supplemented with FGM-2 bullet kit. All cancer cells were from Korean cell line bank (KCLB, Korea). Human colon cancer (SW620) and human gastric cancer (MKN74) were cultured in Dulbescco's modified Eagle's mideiun (DMEM, Hyclone) supplemented with 10% fetal bovine serum (FBS, Gibco), penicillin, and streptomycin (100 U/mL) (Sigma Aldrich). All cells were incubated at 37°C in a humidified 5% CO_2_ atmosphere.

### Tumor Cells Viability, Morphology and Proliferation Assay

Human umbilical vein endothelial cells (HUVECs, Lonza) and red fluorescent protein-expressing HUVECs (RFP-HUVECs) (Angio-Proteomie) were cultured in endothelial basal medium-2 (EBM-2, Lonza) supplemented with EGM-2 bullet kit and normal human lung fibroblasts (LFs, Lonza) were cultured in fibroblast basal medium (FBM-2, Lonza) supplemented with FGM-2 bullet kit. All cancer cells were from Korean cell line bank (KCLB, Korea). Human colon cancer (SW620) and human gastric cancer (MKN74) were cultured in Dulbescco's modified Eagle's mideiun (DMEM, Hyclone) supplemented with 10% fetal bovine serum (FBS, Gibco), penicillin, and streptomycin (100 U/mL) (Sigma Aldrich). All cells were incubated at 37°C in a humidified 5% CO_2_ atmosphere.

For 2D assay, PDMS block was punched using 6 mm biopsy punch and pre-treated with 10 μg/mL of fibronectin (Sigma) for 2 h and washed 3 times before cancer cell seeding. Cancer cells were seeded with 100 μL of the 1.5 × 10^4^ cells/ml density into each well. After 2 days of DMEM media culture, culture media was changed into different contained media with different concentration (0.0%, 0.2%, and 0.4%) and analyzed after 3 days of culture.

### Cancer Cells Migration Assay

Channels configuration is shown on Figure 4A. The upper channel was injected with dissociated LFs (8 × 10^6^ cells/ml). The center channel was filled with acellular HA/fibrin composite of 0.0%, 0.2%, and 0.4% HA, in the different devices. SW620 or MKN74 cancer cells suspended in EGM-2 at a concentration of 10 × 10^6^ cells/ml were introduced at the boundary of center channel. The microfluidic chip was tilted by 90 degrees for 30 min for adhering the tumor cells onto the HA/fibrin composite surface. The devices were filled with EGM-2 media and cells were incubated at 37°C in a humidified 5% CO_2_ atmosphere for 5 days. Number of migrated tumor cells and invasion area were evaluated using ImageJ with the Fiji plugin.

### Angiogenesis Induced by Paracrine Signaling From TME Assay

Channels configuration is shown on Figure 5A. The upper channel were injected with the mixture of HA/fibrin composite (0.0, 0.2, and 0.4% HA), LFs (8 × 10^6^ cells/ml) and SW620 or MKN74 (8 × 10^6^ cells/ml). Briefly, dissociated LFs and SW620 or MKN74 cells in EGM-2 media were mixed at 1:1 volume ratio, before mixing with HA/fibrin. The device was incubated for 24 h at 37°C and 5% CO_2_ to dissipate the air bubbles and to establish the tumor microenvironment within the mineralized matrix by interaction between tumor cells with fibroblasts. Angiogenesis cell seeding was conducted based on the established procedure (Kim et al., 2013). HUVECs suspended in EGM-2 at a concentration of 8 × 10^6^ cells/ml were introduced at the boundary of center channel. The microfluidic chip was tilted by 90 degrees for 30 min for adhering the HUVECs onto surface of the central fibrin gel. The devices were filled with EGM-2 media and cells were incubated at 37°C in a humidified 5% CO_2_ atmosphere. The tumor microenvironment induced angiogenesis assay was conducted for 5 days. Angiogenesis sprouting length was determined using ImageJ with the Fiji plugin.

### Vascularized Three-Dimensional Tumor Spheroid in the HA/Fibrin Composite

Channels configuration is shown on Figure 6A. The upper channel injected with LFs (4 × 10^6^ cells/ml) and fibrin matrix. Three-dimensional SW620 and MKN74 spheroids with LF were prepared using SpheroFilm (INCYTO) (Figure S12). In detail, each SW620 and MKN74 suspension cells (5 × 10^5^ cells/ml; 3.5 ml per 60 mm^2^ dish) were mixed with trypsinized LF (5 × 10^5^ cells/ml; 3.5 ml per 60 mm^2^ dish) at 1:1 ratio and cultured in EGM-2 medium. After 2 days, the media in the dish changed and the spheroids were cultured for 4 days. After 4 days, the spheroids were collected using cell strainer and were mixed with HA/fibrin composite (0.0, 0.2, and 0.4% HA). Angiogenesis cell seeding was conducted based on the established procedure (Kim et al., 2013). HUVECs suspended in EGM-2 at a concentration of 8 × 10^6^ cells/ml were introduced at the boundary of center channel. The microfluidic chip was tilted by 90 degrees for 30 min for adhering the HUVECs onto the HA/fibrin composite surface. The devices were filled with EGM-2 media and cells were incubated at 37°C in a humidified 5% CO_2_ atmosphere. 3D tumor microspheroid with angiogenesis was conducted for 5 days. Number of sprouts and sprouting length were determined using ImageJ with the Fiji plugin.

### Enzyme-Linked Immunosorbent Assay (ELISA)

Twenty-foury transwells were used for medium for further ELISA analysis. The transwell were loaded with the mixture of HA/fibrin composite (0.0, 0.2, and 0.4% HA), LFs (8 × 10^6^ cells/ml) and SW620 or MKN74 (8 × 10^6^ cells/ml). Briefly, dissociated LFs and SW620 or MKN74 in DMEM media were mixed at 1:1 volume ratio, before mixing with HA/fibrin composite prior to loading into the transwell. The transwells were filled with EGM-2 media and were incubated at 37°C in a humidified 5% CO_2_ atmosphere. After 3 days, the medium from each device was collected for ELISA analysis base on standard procedure (Chung et al., 2017).

### Immunostaining and Imaging

For immunostaining, cell samples were prepared followed the standard procedure. The samples were fixed with 4% (v/v) paraformaldehyde in PBS for 10 min, followed by permeabilization for 15 min with a 0.2% Triton X-100 (Sigma) solution and 1htreatment of 3% bovine serum albumin (BSA, Sigma). The samples were stained and incubated at least overnight at 4°C with respective primary antibody as required before stained with Hoechst 33342 (1:1,000) for 1 h of incubation at room temperature on the next day. F-actin, Phalloidin (Alexa Fluor 488) with dilution 1:150 was used. The samples were washed three times and stored in PBS before imaging. For cross-section and whole-construct imaging of 3D blood vessels, stained samples were examined using a FluoView FV1000 confocal laser scanning unit with the IX81 inverted microscope (Olympus). Confocal images were processed by IMARIS software (Bitplane).

### Statistical Analysis

Prism (GraphPad, USA) was used for one-way ANOVA analysis with Tukey's post-test. ^***^ denotes *p* < 0.001, ^**^ denotes 0.001 < *p* < 0.01, ^*^ denotes 0.01 < *p* < 0.05. For significant testing between two conditions non-paired student's *t*-test as used. All data were expressed as the mean ± standard deviation (SD).

## Data Availability

The raw data supporting the conclusions of this manuscript will be made available by the authors, without undue reservation, to any qualified researcher.

## Author Contributions

JA, NJ, and JK conceived and initiated the entire study. JA, NJ, JLim, and JLee conducted microfluidic experiments. T-EP, YK, JK, and NLJ advised the whole microfluidic experiments. JK and NLJ supervised all aspect of this study.

### Conflict of Interest Statement

The authors declare that the research was conducted in the absence of any commercial or financial relationships that could be construed as a potential conflict of interest.
