# Supplementary material for: 3D Microfluidic Bone Tumor Microenvironment Comprised of Hydroxyapatite/Fibrin Composite
Source: Front Bioeng Biotechnol. 2019 Jul 17;7:168. doi: 10.3389/fbioe.2019.00168 (PMC6653063; doi:10.3389/fbioe.2019.00168)
Supplement: Supplementary file 1 [file Data_Sheet_1.PDF]

Reconstitution of the tumor microenvironment in a microfluidic platform comprising a bone-mimetic hydroxyapatite/fibrin composite

Jungho Ahn[1,2,†], Jungeun Lim[1,2,†], Norhana Jusoh[1,†], Jungseub Lee[1], Tae-eun Park[3], YongTae Kim[2,4,5,6], Jangho Kim[7,*] and Noo Li Jeon[1,8,9,10,*]

[1]Department of Mechanical and Aerospace Engineering, Seoul National University, Seoul 08826, Republic of Korea

[2]George W. Woodruff School of Mechanical Engineering, Georgia Institute of Technology, Atlanta, GA 30332, USA

[3]Ulsan National Institute of Science and Technology, Ulsan 44914, South Korea

[4]Parker H. Petit Institute for Bioengineering and Bioscience, Georgia Institute of Technology, Atlanta, GA 30332, USA

[5]Institute for Electronics and Nanotechnology, Georgia Institute of Technology, Atlanta, GA 30332, USA

[6]Wallace H. Coulter Department of Biomedical Engineering, Georgia Institute of Technology, Atlanta, GA 30332, USA

[7]Department of Rural and Biosystems Engineering, Chonnam National University, Gwangju, 500-757, South Korea

[8] Division of WCU (World Class University) Multiscale Mechanical Design, Seoul National University, Seoul 08826, Republic of Korea

[9] Seoul National University Institute of Advanced Machines and Design, Seoul 08826, Republic of Korea

[10] Institute of Bioengineering, Seoul National University, Seoul, Republic of Korea

*** Correspondence:**
Corresponding Author
njeon@snu.ac.kr

† Equally Contributed: These authors contributed equally

Keywords: Tumor microenvironment, Vascularized tumor, Hydroxyapatite, Fibrin matrix

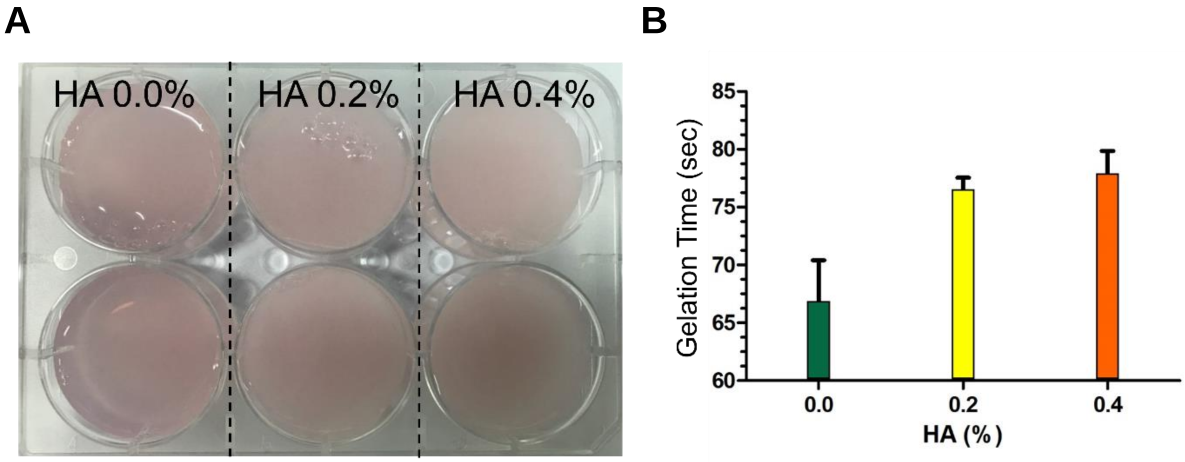

Figure S1. Gelation time for HA/fibrin composite with each concentration of HA. (A) HA/fibrin composite in 6-well plate with 0.0, 0.2 and 0.4% HA contents respectively. (B) Time taken to gelate completely after reacting with thrombin with varying concentration of HA components (0.0, 0.2 and 0.4 %).

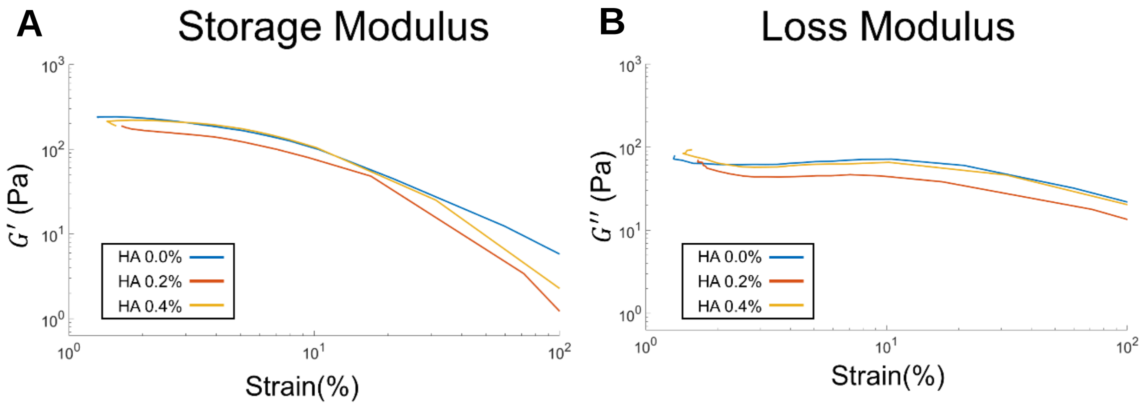

Figure S2. Gel rheology analysis as a function of strain for HA/fibrin composite with each concentration of HA. The (A) storage and (B) loss modulus as a function of strain at the same temperature and oscillation frequency in gel rheology analysis for HA/fibrin composite with varying concentration of HA contents (0.0, 0.2 and 0.4 %).

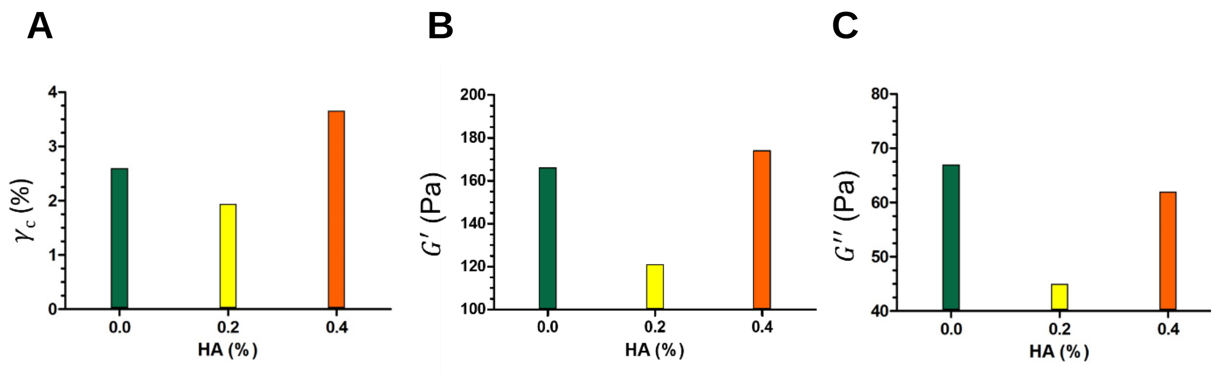

Figure S3. Critical strain and value of modulus of HA/fibrin composite for each HA concentration. (A) The critical strain of HA/fibrin composite for each concentration of HA. The (B) storage and (C) loss modulus at 5% strain state in gel rheology analysis for HA/fibrin composite with varying concentration of HA components (0.0, 0.2 and 0.4 %).

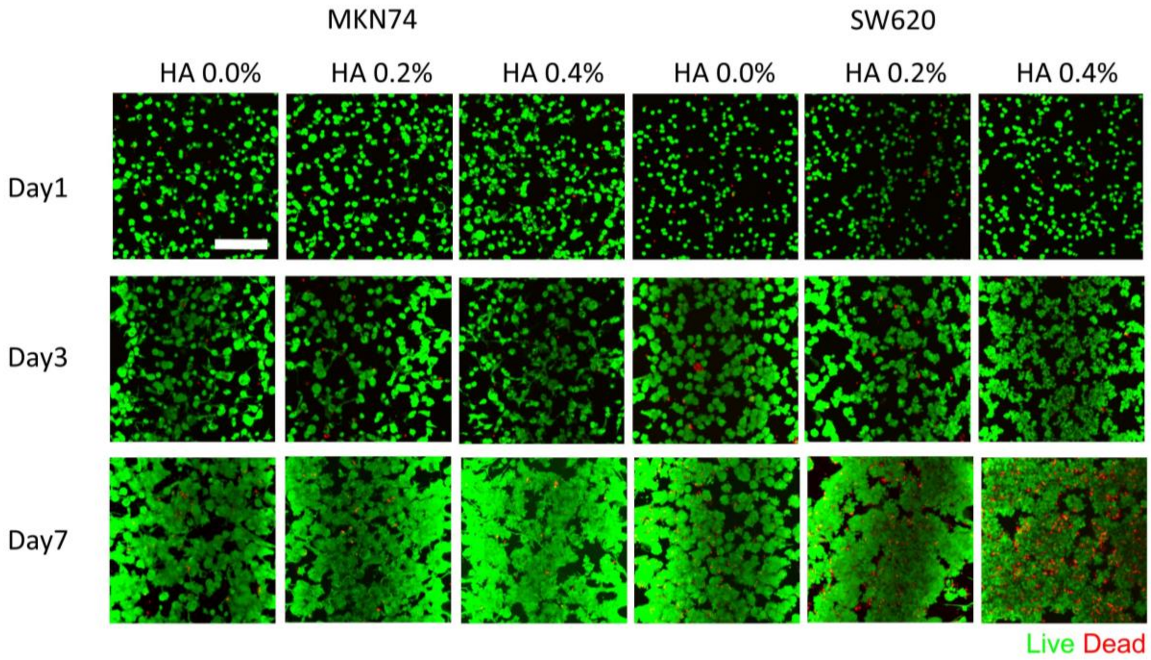

Figure S4. Confocal live/dead images of MKN74 and SW620 respectively in different HA concentration and culture time points. (green: calcein-AM, red: ethd-1) (n= 4-6 chips per condition) (Scale bar: 200 µm)

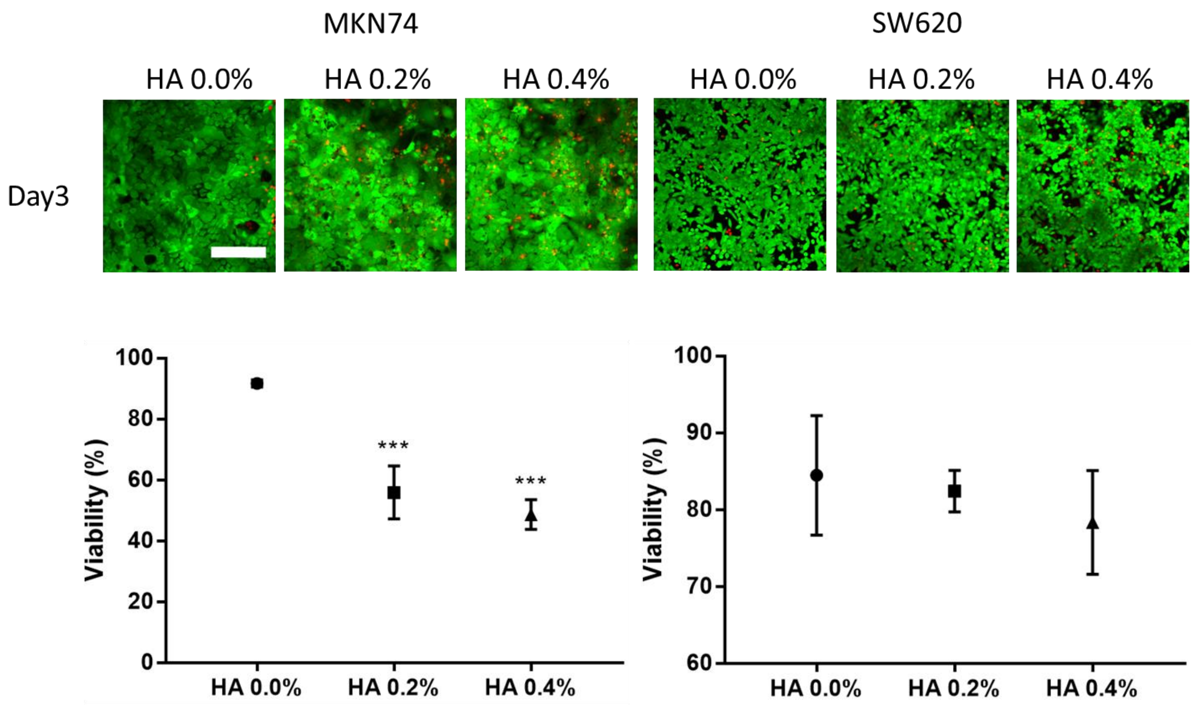

Figure S5. Tumor cell viability in 2D culture with varying HA concentration.

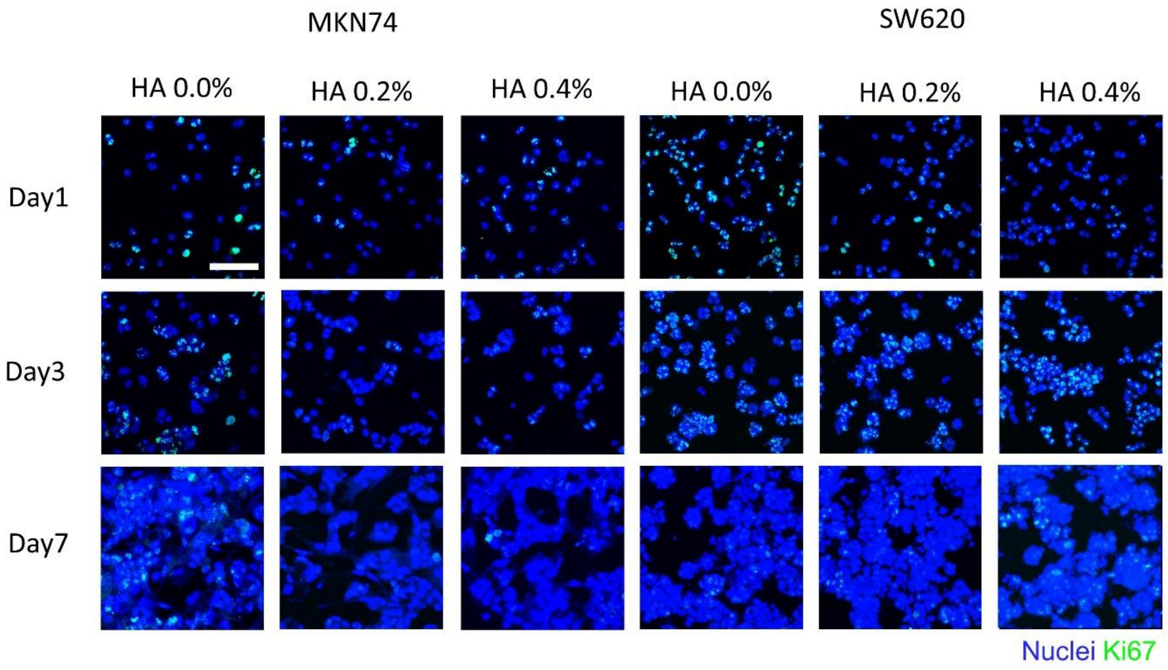

Figure S6. Confocal images for cell proliferation of MKN74 and SW620 respectively in different HA concentration and culture time points. (green: Ki-67, blue: nuclei) (n= 4-6 chips per condition) (Scale bar: 200 μm)

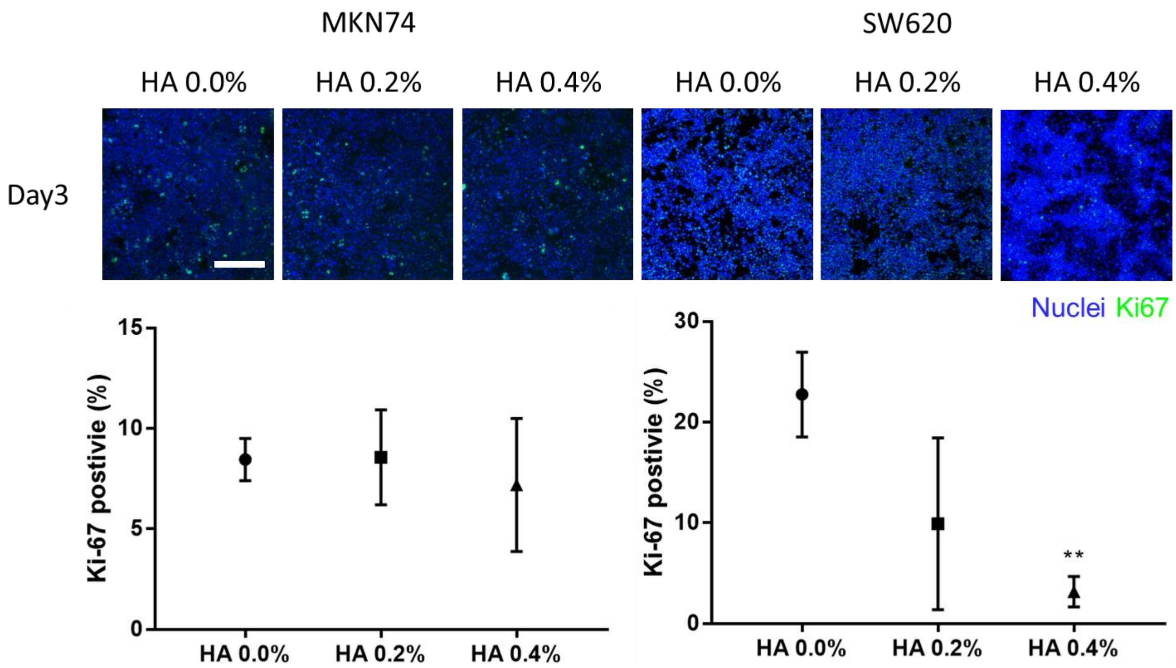

Figure S7. Tumor cell proliferation in 2D culture with varying HA concentration. (Scale bar: 200 μm)

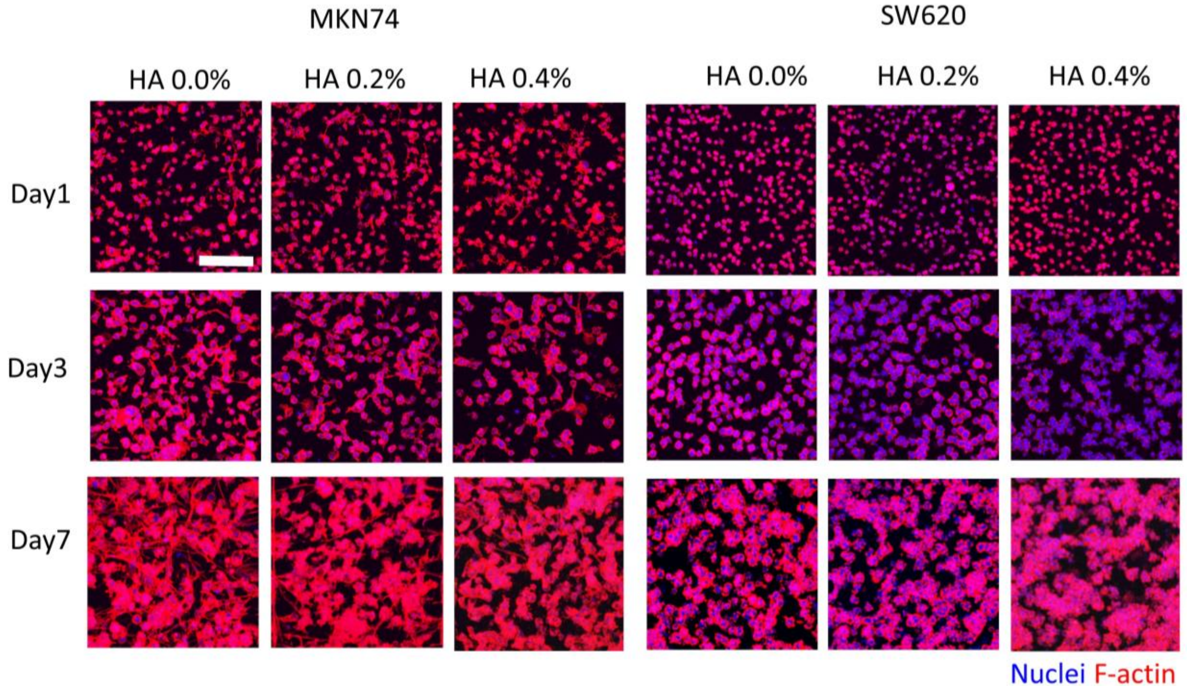

Figure S8. Confocal image analysis of tumor cell morphology of MKN74 and SW620 respectively in different HA concentration and culture time points. (red: F-actin, blue: nuclei) (n= 4-6 chips per condition) (Scale bar: 200 μm)

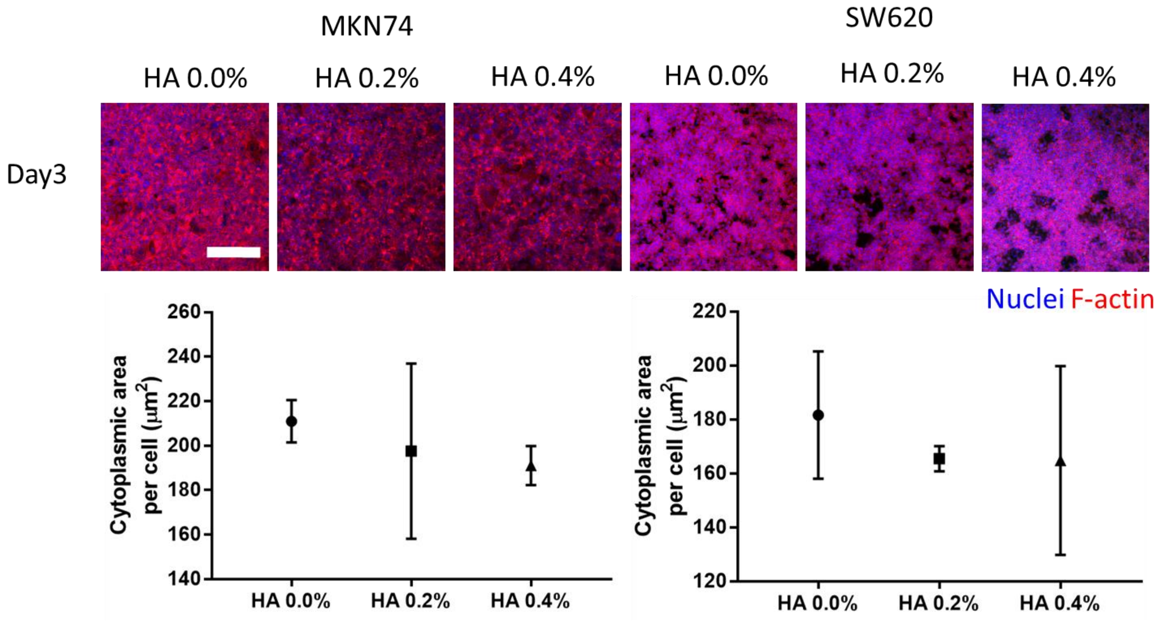

Figure S9. Tumor cell cytoplasmic area in 2D culture with varying HA concentration. (Scale bar: 200 μm)

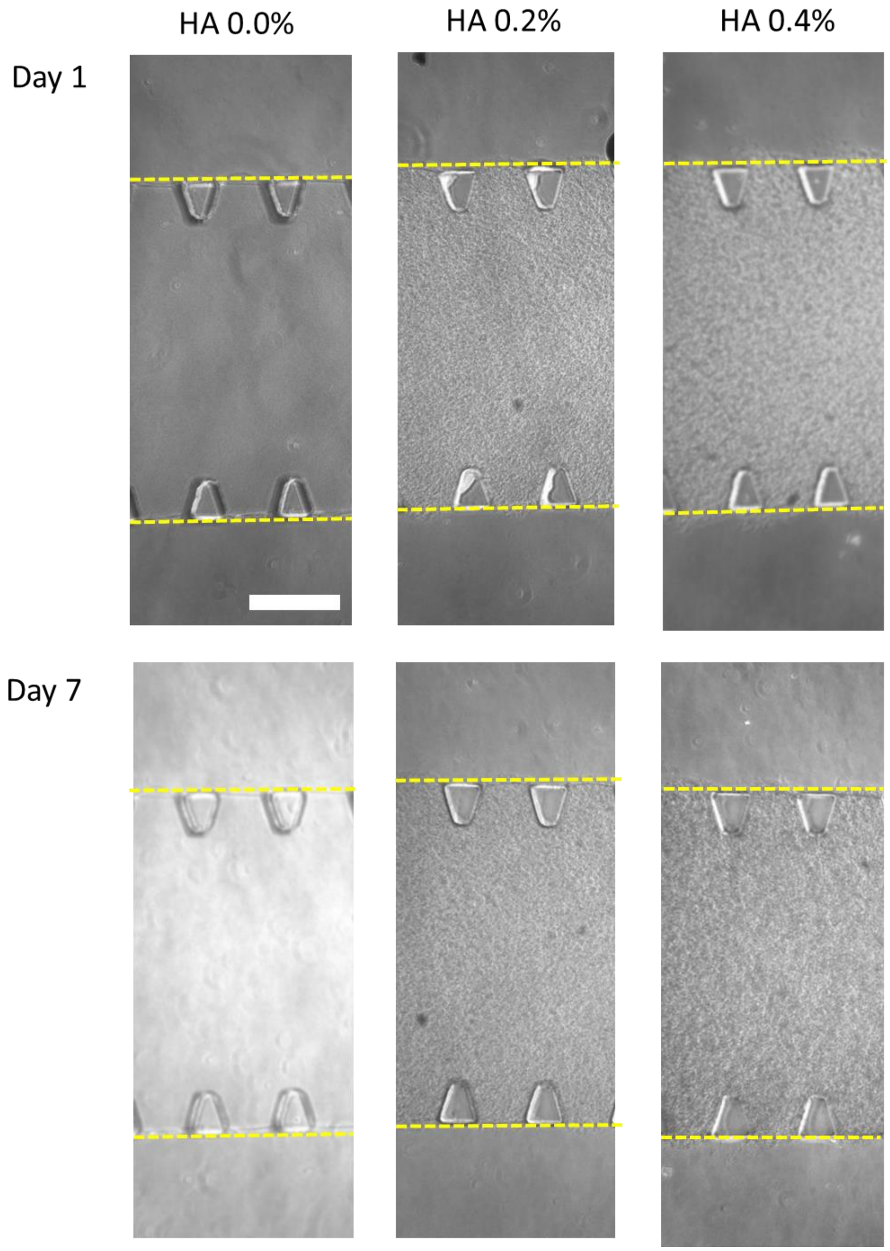

Figure S10. Gel construct observation under experiment conditions. HA-fibrin composite with varying HA concentration (0.0%, 0.2%, and 0.4%) was filled into middle channel and no cells were injected to other side channels. Scale bar: 200 μm

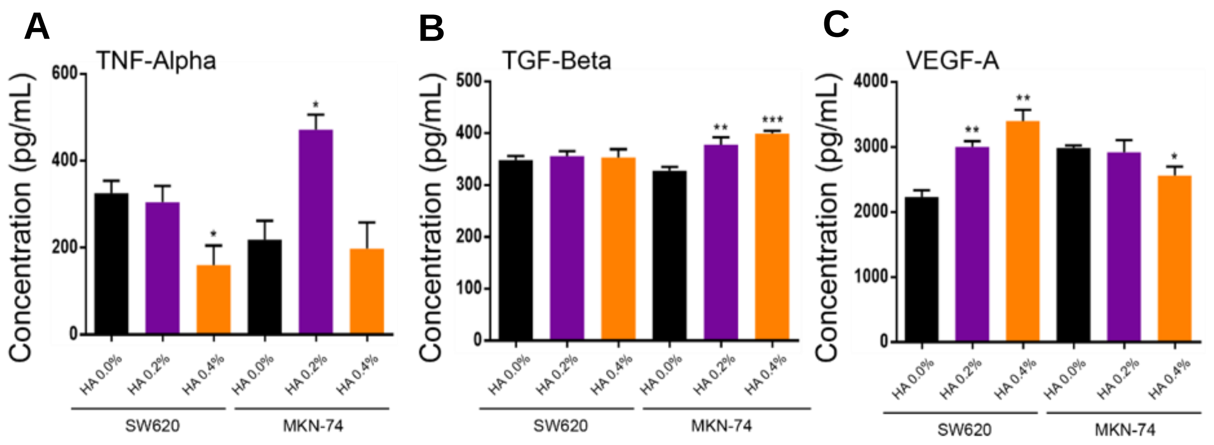

Figure S11. ELISA quantification of growth factors/cytokines. (A) TNF-α (B) TGF-ß (C) VEGF-A for each concentration of HA (0.0, 0.2 and 0.4 %) in HA/fibrin composite.

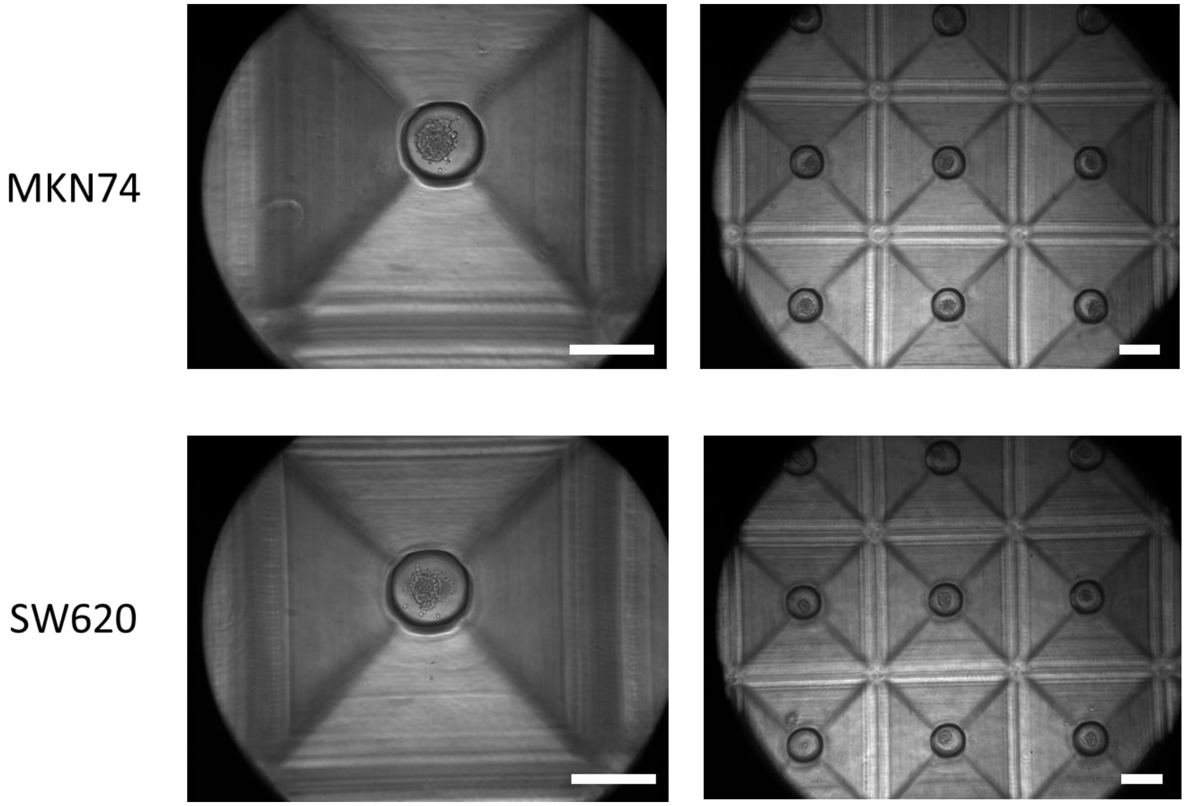

Figure S12. Tumor-fibroblast microspheroid formation using SpheroFilm(INCYTO) at day 2. Scale bar: 300 μm

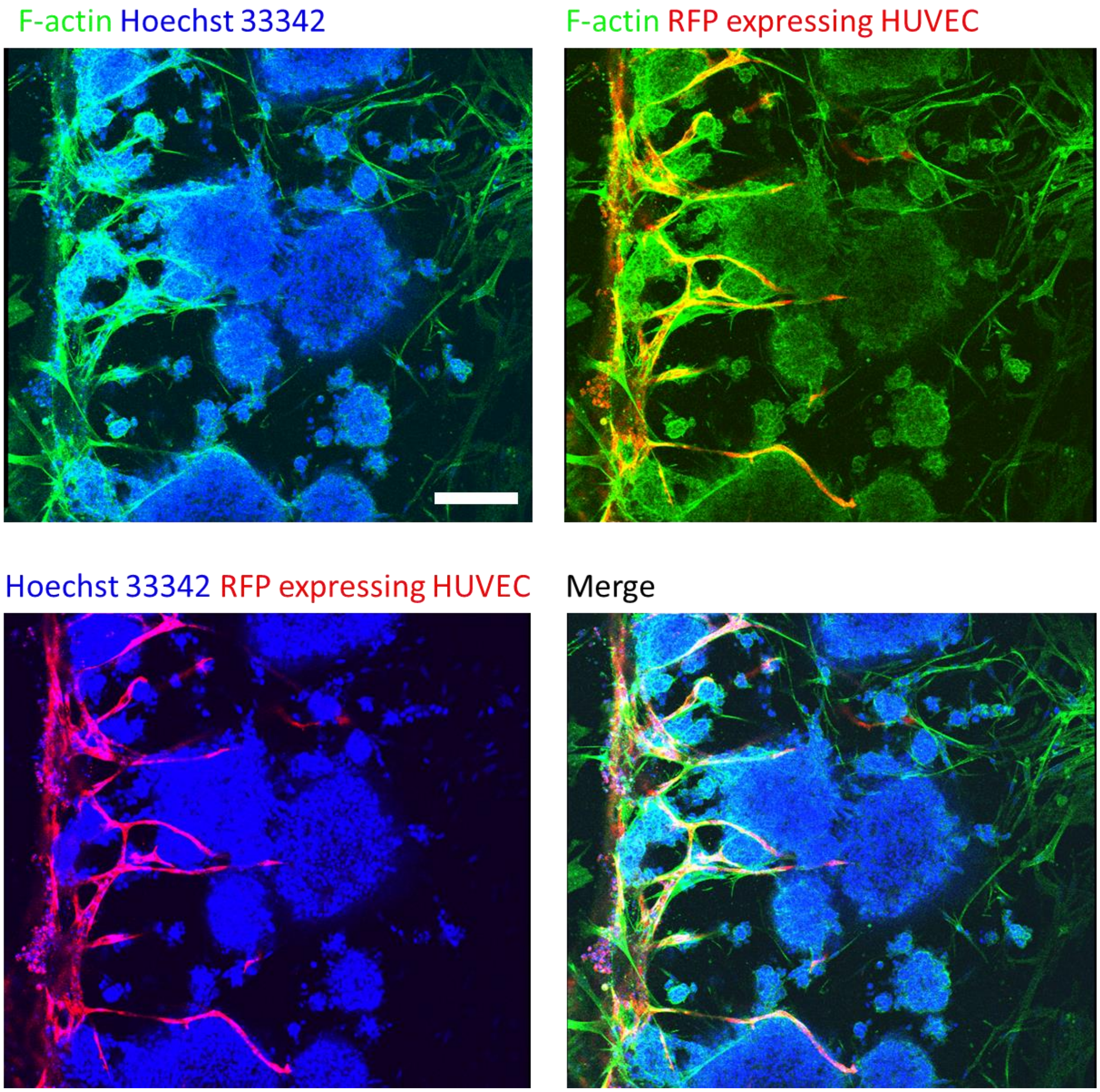

Figure S13. Representative confocal images of direct interaction between tumor-fibroblast microspheroid and blood vessel. Scale bar: 100 μm.

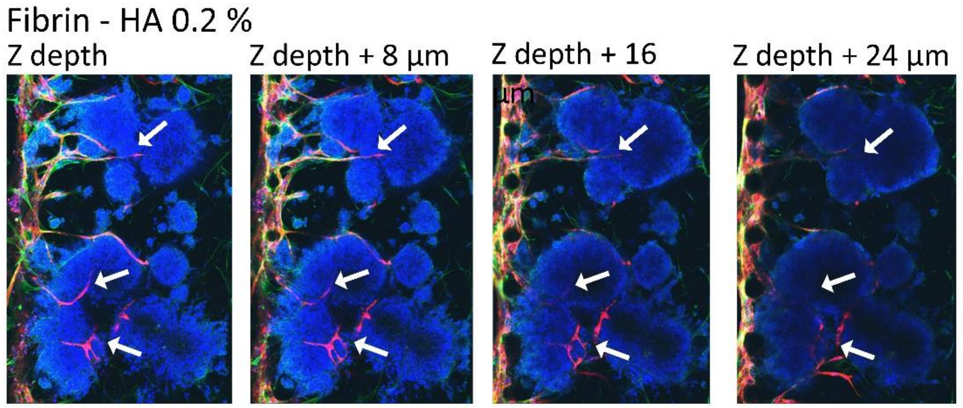

Figure S14. Representative confocal z-section images of direct interaction between blood vessel and tumor microspheroid (white arrows indicate direct interaction between endothelial cells and the microspheroids). (Scale bar: 200 µm)
